# Longitudinal impacts of an online safety and health intervention for women experiencing intimate partner violence: randomized controlled trial

**DOI:** 10.1186/s12889-020-8152-8

**Published:** 2020-02-26

**Authors:** Marilyn Ford-Gilboe, Colleen Varcoe, Kelly Scott-Storey, Nancy Perrin, Judith Wuest, C. Nadine Wathen, James Case, Nancy Glass

**Affiliations:** 1 Arthur Labatt Family School of Nursing, University of Western Ontario, FNB 2302, 1151 Richmond St, London, ON N6A 5C1 Canada; 2 School of Nursing, University of British Columbia, Vancouver, BC Canada; 3 Faculty of Nursing, University of New Brunswick, Fredericton, NB Canada; 4 School of Nursing, Johns Hopkins University, Baltimore, MD USA; 5 Faculty of Information and Media Studies, University of Western Ontario, London, ON Canada

**Keywords:** Intimate partner violence against women, Randomized controlled trial, Complex interventions, E-health, Safety planning, Mental health, Technology, Mastery, Self-efficacy, Coercive control

## Abstract

**Background:**

Responding to intimate partner violence (IPV) and its consequences is made complex by women’s diverse needs, priorities and contexts. Tailored online IPV interventions that account for differences among women have potential to reduce barriers to support and improve key outcomes.

**Methods:**

Double blind randomized controlled trial of 462 Canadian adult women who experienced recent IPV randomly were assigned to receive either a *tailored*, interactive online safety and health intervention (*iCAN Plan 4 Safety*) or a static, *non-tailored* version of this tool. Primary (depressive symptoms, PTSD symptoms) and secondary (helpfulness of safety actions, confidence in safety planning, mastery, social support, experiences of coercive control, and decisional conflict) outcomes were measured at baseline and 3, 6, and 12 months later via online surveys. Generalized Estimating Equations were used to test for differences in outcomes by study arm. Differential effects of the tailored intervention for 4 strata of women were examined using effect sizes. Exit survey process evaluation data were analyzed using descriptive statistics, t-tests and conventional content analysis.

**Results:**

Women in both tailored and non-tailored groups improved over time on primary outcomes of depression (*p* < .001) and PTSD (*p* < .001) and on all secondary outcomes. Changes over time did not differ by study arm. Women in both groups reported high levels of benefit, safety and accessibility of the online interventions, with low risk of harm, although those completing the tailored intervention were more positive about fit and helpfulness. Importantly, the *tailored* intervention had greater positive effects for 4 groups of women, those: with children under 18 living at home; reporting more severe violence; living in medium-sized and large urban centers; and not living with a partner.

**Conclusion:**

This trial extends evidence about the effectiveness of online safety and health interventions for women experiencing IPV to Canadian women and provides a contextualized understanding about intervention processes and effects useful for future refinement and scale up. The differential effects of the tailored intervention found for specific subgroups support the importance of attending to diverse contexts and needs. *iCAN* is a promising intervention that can complement resources available to Canadian women experiencing IPV.

**Trial registration:**

Clinicaltrials.gov ID NCT02258841 (Prospectively Registered on Oct 2, 2014).

## Background

Intimate partner violence (IPV) is a complex public health and human rights issue that affects 1 in 3 women globally from all social, economic and cultural groups [1]. The negative effects of IPV are broad and often linked, impacting women’s safety, mental and physical health, social relationships, economic situation, and parenting [2–7]. For example, the chronic stress of experiencing IPV has been found to erode women’s mental health, with depression and PTSD being common, often long-term, problems for women [8]; concurrently, poorer mental health has been associated with other challenges, including difficulty maintaining separation from an abusive partner [9]. If, when and how women seek help or attempt to deal with the violence and its effects is often a long-term process shaped by relationship dynamics and diverse priorities, needs and conditions [10–13]. As such, women in unsafe intimate relationships are most likely to benefit from interventions that consider the context and complexity of their lives and that are personalized or tailored to their unique circumstances, priorities and needs. Importantly, evaluations of ‘complex’ interventions should examine more than ‘main effects’ but should also assess differential impacts across subgroups (attending to differences among women) and explore who, how and why expected changes occur or do not occur [14]. This approach is needed to develop a contextualized understanding of intervention effectiveness while producing insights useful for successful implementation and scale up.

Relatively few interventions have been shown to improve the safety, health or quality of life of women experiencing IPV, although there is growing evidence that some types of face-to-face interventions, including advocacy and cognitive behavioural therapy, are effective with some populations and/or under certain conditions [15–17]. Interest in developing online interventions for women experiencing IPV has recently emerged, in part, because of their potential to be tailored and to reduce practical or perceived barriers to assistance, such as lack of services, a desire for privacy, or stigma [18]. Thus, online interventions have potential to reduce inequities among women who face the most substantial barriers to support, including Indigenous, racialized and/or immigrant women, those living in rural communities, and women with partners other than men [19–21]. Effective e-health interventions often integrate interaction, feedback and tailoring as key features and exist in a number of areas, including mental health, sexual health and smoking [22–24]. However, few such interventions have been developed and tested among women experiencing IPV. If effective, tailored online interventions could offer a relatively inexpensive strategy for improving women’s awareness of their safety risks and options, and enhancing their sense of control, confidence, and mental health – factors that are often eroded by IPV but that are critical to women’s ability to lead safer, more satisfying and productive lives [7].

This research is part of an international collaboration of teams in the United States (US), New Zealand (NZ), Australia and Canada testing country-specific versions of an online intervention for women experiencing IPV in randomized controlled trials employing similar methods and outcomes [25–28]. Beginning with a foundational online safety decision aid developed in the United States [29], teams in NZ, Australia and Canada adapted and extended this intervention to fit with their particular contexts. Each team drew on additional theories, research and stakeholder consultations to frame their adaptations and selectively added new features. In developing the Canadian version - *iCAN Plan 4 Safety (iCAN)*, we drew on principles of trauma- and violence-informed care (TVIC) [30, 31] to prioritize women’s physical and emotional safety, choice and control, and to emphasize inclusiveness, particularly for Canadian women who face barriers to support, including those no longer living with an abusive partner [26]. Drawing on substantial research on the health effects of IPV [3, 32, 33], including our own work [34–36], we added new strategies to explicitly address aspects of women’s health and well-being, including approaches for managing distressing mental and physical health problems, and added a debriefing activity at the end of the tool [26].

In trials completed in the US (IRIS) [37], NZ (I-SAFE) [38] and Australia (I-DECIDE) [39], women in both study arms (tailored online intervention, general information) improved over time on most primary and secondary outcomes. However, between-group differences were only found in the US-based IRIS study, where the tailored intervention was more effective than general online information in reducing *decisional conflict* after one use of the tool and in increasing the *use of helpful safety actions* over a 12-month period [37]. In each of these 3 studies, women reported that the tailored online intervention was acceptable and helpful to them, with no evidence of harms.

In New Zealand, I-SAFE was developed with the intention of being *inclusive* and *appropriate* for both Maori and non-Maori women [28]. Indeed, results of the I-SAFE trial underscore the importance of considering the differential effects of these types of complex, online interventions. Specifically, Maori women were more likely to benefit from the tailored intervention in terms of reductions in both *depression* and *severity of violence* (primary outcomes), an important finding given Maori women’s increased risk of violence compared to the NZ population of women, and the considerable barriers they face to obtaining support [38]. Like I-SAFE, we developed *iCAN* with an explicit aim of ensuring inclusiveness and fit for diverse groups of women [26].

### Objectives and hypotheses

The primary aim of this study was to test the effectiveness of *iCAN,* an interactive, tailored, online safety and health intervention on mental health and safety outcomes of Canadian women experiencing IPV. We compared the tailored, interactive intervention with a non-tailored version that was brief and static. We hypothesized that the tailored version would reduce symptoms of depression and posttraumatic stress disorder (PTSD; primary outcomes) and improve women’s confidence in safety planning, mastery, safety behaviors, social support, experiences of coercive control and decisional conflict (secondary outcomes). These secondary outcomes are linked directly to the content of the intervention and understood to be mechanisms that could explain how *iCAN* might improve women’s mental health.

Consistent with guidelines for testing complex interventions [14], we also examined the differential effects of the tailored and non-tailored versions for specific groups of women identified a priori [26]. Furthermore, we conducted a concurrent process evaluation, drawing on both quantitative and qualitative data, to assess women’s perceptions of use, acceptability, helpfulness and potential harms of both versions in an effort to better understand what might account for any intervention effects. As such, the *iCAN* trial builds on and extends the approaches used in the other trials by seeking to further contextualize and explain the impacts of the online intervention, drawing on a combination of subgroup analysis and a comprehensive process evaluation. In this manuscript, we focus on the analysis of primary and secondary outcomes by study arm and the subgroup analyses. We briefly present selected findings from the process evaluation based on the exit survey data in order to contextualize these results. However, analysis of the qualitative interview data is presented in detail elsewhere [40].

## Method

### Trial design

We conducted a double-blind, parallel, randomized controlled trial (RCT) from October 2014 to January 2017. Using 1:1 allocation, women were randomly assigned to receive *iCAN*, an interactive, *tailored* online safety and health intervention or a brief, static version that was *not* tailored (i.e., not personalized). Given the heightened risk of harm and poor health among women experiencing IPV, designing the trial to avoid further harms was a priority. We intentionally selected a brief, non-tailored version of the tailored intervention as the comparison condition (rather than a true control) as a means of promoting women’s safe participation in the study (regardless of study arm), since providing basic information about abuse and available services to support safety planning is part of usual care and this information is widely available to women online. The study protocol (ClinicalTrials.gov identifier NCT02258841) was developed using CONSORT guidelines for RCTs [41] and CONSORT e-health guidelines [42]. Ethics approval for this study was obtained in July 2014 from the Institutional Research Ethics Boards at the University of Western Ontario, University of British Columbia, and University of New Brunswick. Details of the study protocol are provided elsewhere [26].

### Participant enrollment and randomization

Participation was open to adult (19 years or older), English-speaking women living in 3 provinces (British Columbia, Ontario, New Brunswick) who reported that they had experienced IPV in the previous 6 months. Women who had separated from an abusive partner were eligible if the separation had occurred in the previous 12 months. To participate, women also needed a safe computer to access the online intervention, a safe email address to receive study information, and a secure mailing address for receiving study honoraria. The power analysis was based on baseline means and standard deviations for depression and PTSD from the IRIS trial [37]. We planned to recruit a sample of 450 women (225 per group), assuming 10% attrition and based on the ability to detect a 15–20% difference in the primary outcomes (depression and PTSD) across groups with statistical power of 0.80 and alpha of 0.05.

Details of participant recruitment and enrollment can be found elsewhere [26]. Briefly, participants were recruited primarily using online advertisements, supplemented by flyers posted in community settings (such as libraries) or through organizations or agencies serving women. Potential participants were directed to the study website for more information. Those who were interested in enrolling contacted a Research Assistant (RA) using a toll-free telephone number for eligibility screening, verbal consent, and enrollment. To enroll eligible women, RAs entered information about women’s safe contact information into a secure online tracking database. For each woman, this database automatically generated a unique study ID, randomized the participant to group, and sent an email message containing a link to the study Letter of Information and Consent, a user name and password, a URL for the password-protected online intervention to which she had been assigned, and information about safe access to the website and how to obtain technical support if needed.

To achieve balance in the sample across the study sites, a stratified block randomization scheme was used based on both the province of residence and whether the woman had children under 18 years living at home. The randomization algorithm was pre-programmed into the study tracking database by the study programmer who had no contact with participants. Participants were not informed of their group assignment. The research team members other than the programmer (JC) and statistician (NP), were blind to group assignment until the final 12-month surveys had been completed.

### Procedures

After enrollment, women used the URL and login credentials provided to them to confirm their consent, to complete the study measures, and then access the online intervention at their convenience and when they deemed it was safe. Automated and manual messages from RAs were sent at regular intervals to encourage completion of the baseline measures until the 6-week enrollment period closed. Those who completed the baseline survey were sent reminder messages to complete 3-, 6- and 12-month follow up surveys at regular intervals until the survey was completed or the 6-week time frame for completion ended. Participants were provided honoraria (mailed or electronic gift cards) when completing up to 4 surveys, with the amount increasing incrementally at each time point ($20, $30, $40, $50). The assigned online intervention was available to women for the full 12-month period of the trial. Recruitment opened in October 2014 and was completed in December 2015.

At the end of the 12-month survey, participants received a brief exit survey asking for feedback on acceptability, safety, harms and helpfulness of the online intervention they completed. They were also asked about their interest in completing a qualitative telephone interview about their experiences of the intervention and the study. The trial ended when the last 12-month survey had been completed in January 2017. In a separate phase, in-depth qualitative telephone interviews with a trained RA or investigator were conducted with a sub-sample of 52 women and completed in April 2017, the results of which are reported elsewhere [40].

Women’s safety was prioritized in designing all aspects of this study [26]. The websites housing the surveys and interventions were designed with quick escape buttons and information about how to access the sites in private mode. Research staff received training in safety assessment and referral and use of a standard safety protocol to guide all interactions with participants. The language and content of surveys and the interventions were carefully drafted to increase women’s comfort and emotional safety and to convey inclusiveness for participants of diverse backgrounds and varied types of relationships. An independent Data Safety Monitoring Committee met approximately every 6 months to review safety outcomes.

### Interventions

For detailed descriptions of the tailored and non-tailored interventions, see the protocol [26]. Key features of each intervention are summarized and compared in Table 1. Briefly, in both study arms, women were initially asked to respond to background questions about their demographic characteristics, living situations and their plans for their relationship with the abusive partner (i.e., planning to stay, leave, remain separated, return to partner or unsure). In the tailored intervention group, women engaged in interactive activities designed to increase their awareness of safety risks and reflect on their plans for their relationships and priorities. They completed the Danger Assessment tool [43] and received immediate feedback on their level of risk. Next, they rated the relative importance of 5 factors (i.e. safety concerns, child well-being, health and well-being, having resources, feelings for partner, organized in pairs) in making decisions about their unsafe relationship; a graph showing the ranked importance of these priorities was presented to the woman (based on her ratings), along with suggestions for strategies that fit with her top priority. Finally, each woman was provided with a *personalized* detailed action plan of strategies and resources for addressing their safety and health concerns based on responses to background questions and activities, with the option to modify and further personalize the plan if they wished. Messages were carefully written to acknowledge and respect differences among women, and to encourage women to use the information provided in ways that were right for them. In contrast, women in the non-tailored group received general (static) information about the importance of considering priorities when making decisions along with risk factors for IPV; they were provided with a brief standardized action plan focusing on emergency safety planning and child safety strategies and resources only, with no opportunity to modify or personalize the plan. At the end of the online intervention, women in both groups received standardized debriefing information about symptoms of a stress reaction and strategies to manage these.

**Table 1:** Active Components of the Tailored and Non-Tailored Online Interventions

Component	Intervention	
	Tailored Intervention	Non-Tailored
Priorities	• Interactive priorities exercise • Personalized feedback about the woman’s ‘top’ priority’ and recommendations for related information in the action plan	• Brief statement about the importance of women’s priorities to decision-making
Risk Assessment	• Completion of the Danger Assessment Calendar and Questions with personalized feedback	• Brief general information about risk factors for IPV
Action Plan	• 54 Strategies organized in 8 categories • Resources (contact information for services or helpful websites) associated with most strategies • Specific strategies recommended based on the woman’s responses to background questions and results of priority exercise and risk assessment; • Woman can modify the plan as she chooses	• 10 strategies focussed on emergency safety planning; • Selected resources provided for crisis services only • No recommendations based on the woman’s situation; • No opportunity to modify the plan

### Outcomes

Primary and secondary outcomes were assessed at baseline (pre-intervention) and 3, 6 and 12 months later via online surveys that women completed when they first opened the link to their assigned intervention website (tailored and non-tailored). One outcome, *decisional conflict*, was measured twice (at baseline and immediately post-intervention).

#### Primary outcomes

*Depressive Symptoms* were measured using the total score on the Center for Epidemiologic Studies Depression Scale, Revised (CESD-R) [44], a 20-item self-report measure of symptoms reflective of the DSM-V criteria for depression. Women rated their symptom frequency in the past week on a 4-point scale (1 = rarely or none of the time to 4 = most of the time), with responses summed produce total scores (range 0–60). Scores ≥22 are consistent with significant clinical depression, while scores between 16 and 21 are consistent with mild to moderate symptomology. Cronbach’s alpha reliability was 0.95 in this sample. *PTSD symptomology* was measured using the total score on the PTSD checklist, Civilian Version (PCL-C), a 17-item self-report measure designed to assess PTSD symptomology in community samples [45]. Women indicated how much they had been bothered by each symptom over the past month using a 5-point (1–5) scale, ranging from 1 (not at all) to 5 (extremely). Total summed scores range from 17 to 85, with a higher score indicating greater symptomatology. Cronbach’s alpha reliability was 0.93 in this sample.

#### Secondary outcomes

*Decisional Conflict* was measured using an adapted 13-item version of the low literacy Decisional Conflict Scale (DCS) [46]. The DCS assessed women’s perspectives of the advantages and disadvantages of safety planning decisions with four subscales: information, values clarity, support, and uncertainty [47]. Summed scores reflect higher levels of Decisional Conflict [46]. Cronbach’s alpha was 0.87 for the total score in this sample. *Helpfulness of Safety Actions* was measured using 22 items adapted from several sources [48, 49]. Women indicated whether they had used each safety action in the previous 12 months (yes/no) and, if used, how helpful this strategy was in dealing with the violence (on a 5-point scale ranging from ‘not at all helpful’ to ‘very helpful’). A total score is the mean helpfulness across the items (Cronbach’s alpha = 0.75). *Mastery,* a person’s perception of the degree of control they have in their lives, was measured using Pearlin’s 7-item Mastery Scale (Cronbach’s alpha = 0.84). Total scores are created by summing responses to all items such that higher scores reflect greater mastery [50–52]. *Self-efficacy for Safety Planning* was measured using visual analogue scales (VAS) developed for this study. Women rated their confidence in making a safety plan for themselves on a 100 mm horizontal line, with anchors of ‘not at all confident’ and ‘completely confident”. Women with children rated their confidence in making a safety plan for their children on a second scale with the same format. VAS scores were recorded by the website as the distance in mm from the left anchor (0) to the location of the mark on the line (range 0 to 100). Higher scores reflect greater self-efficacy for safety planning. *Social Support* was measured using a 5-item version of the Medical Outcomes Study Social Support Survey (MOS-SSS) that assesses perceived availability of emotional, informational, and instrumental support (Cronbach’s alpha = 0.86). Items are rated on a 5-point Likert-type scale, ranging from 1 (none of the time) to 5 (all of the time). Total summed scores are computed, with higher scores suggestive of greater perceived support [53]. *Experiences of Coercive Control* were measured on the 10-item Women’s Experiences with Battering (WEB) Scale [54]. Women rated their agreement with each item on a 6-point Likert scale, ranging from 1 (Strongly agree) to 6 (Strongly disagree). Higher total summed scores reflect greater current impacts of coercive and controlling behavior on the woman (Cronbach’s alpha =0.87).

#### Moderators

*IPV severity* was measured using the 30-item Composite Abuse Scale (CAS) [55]. Women rated the frequency of each abusive act experienced from a partner in the previous 12 months on a 6-point scale ranging from ‘never’ (0) to ‘daily’ (5). In this study, the 3 sexual abuse items were modified to make them more consistent with current theory and measurement approaches in the field [56]. Using established cut scores, women’s responses can be categorized as positive or negative for 4 types of abuse: physical abuse, emotional abuse, harassment, severe combined abuse. A total summed score can also be computed, where higher scores indicate more severe abuse [57]. Cronbach’s alpha was 0.95 for the total score in this sample. *Partner Status* was measured using women’s reports of whether they were living with their abusive partner (yes, no). Whether women had *children under the age of 18 living at home* (yes/no) was asked on the baseline survey. *Geographic Location* was assigned by classifying women’s reports of their community of residence into 3 different types of population centers [58]: large population center (large urban center with a population of 1 million or more), medium population center (medium-sized city, population 30,000 to 999,999), small population center and/or rural area (population less than 29,999).

#### Process evaluation indicators

In the 12-month exit survey, women were asked to rate the acceptability, safety and helpfulness of the online tool using 5-point response options ranging from ‘strongly disagree’ (1) to ‘strongly agree’ (5). Items were drawn from previous studies of IPV interventions [59, 60] and from a version of the Preparation for Decision-Making Scale [61] where women were asked to report on the helpfulness of the online interventions in supporting their efforts to deal with the violence. An open text box was provided to collect any additional comments women wished to share about their participation in the study.

### Data analysis

#### Examination of outcomes by study arm

The effectiveness of the intervention was assessed by comparing the tailored and non-tailored groups on changes in primary and secondary outcomes, between the baseline and 3, 6, and 12 months later, using intent-to-treat principles with Generalized Estimating Equations (GEE). Separate analyses were conducted for each outcome. The parameter of interest was the group (tailored vs. non-tailored) by time interaction, which, if significant, means that change over time differs for tailored and non-tailored groups. The overall effect sizes, for specific outcomes, of the tailored intervention were estimated using Cohen’s d.

#### Analysis of differential intervention effects

We tested for differences in the intervention effects for 4 specific subgroups of women identified using baseline data for: partner status (living with or separately from the partner), whether women had children under the age of 18 living at home (yes/no), severity of IPV (more/less severe, using the median score on the Composite Abuse Scale), geographic location (large urban center, medium-size city, small population center/rural areas). We planned to examine group differences based on Indigenous identification (yes/no) but the number of Indigenous participants (*n* = 62, 13.4%) was too small. Given that these subgroup analyses are not fully powered, we have interpreted differences in effects sizes (Cohen’s d) across the specific subgroups rather than rely on statistical significance.

#### Process evaluation

Descriptive statistics were used to summarize responses to each item and t-tests used to compare women’s ratings of the online intervention by group (tailored, non-tailored). Optional open-ended comments from the 12 month exit survey were summarized using conventional content analysis techniques [62]**.**

## Results

**Fig. 1:**
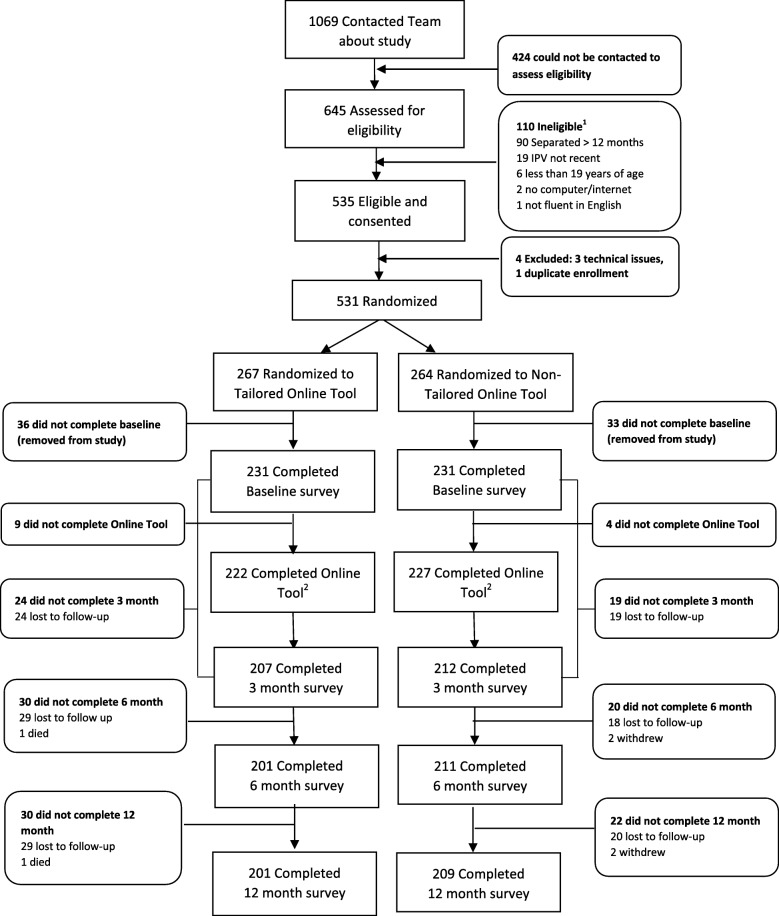
Consort Diagram. ^1^ Total does not equal 110 as some women were ineligible for more than 1 reason. ^2^ “Completed online tool” is defined as working through the tool including the final debriefing page. Stopping at any time before this point is defined as ‘not completing’ the online tool

Of the 1069 women who contacted the study for information about participation, 424 (39.6%) could not be reached to assess them for eligibility. In total, 645 women were assessed for eligibility; of these, 535 (83.0%) were deemed eligible, while 110 women were ineligible, largely (*n* = 90) because they had been separated from their abusive partner for more than 12 months (See Fig. 1). In all, 531 women (99.3% of those eligible) consented to participate and were randomized to either the tailored (*n* = 267) or non-tailored intervention (*n* = 264). Overall, 84.6% (*N* = 231) of participants in the tailored group and 86.5% (*N* = 231) in the non-tailored group completed the baseline survey and were included in the analysis (*N* = 462). Retention was 89.6, 87.0, and 87.0% at 3-, 6-, and 12-months, respectively for the tailored group. In the non-tailored group, retention was 91.8, 91.3, and 90.5% at 3-, 6-, and 12-months, respectively. Attrition across all time points was small and largely due to losing contact with women. No serious adverse events were identified in the conduct of this trial.

Table 2 presents the sample characteristics. The average age of participants was 34.61 years with the majority (70.1%) completing at least some post-secondary school. Almost half (47.0%) reported finding it very or extremely difficult to live on their current income, while another 46.5% found it somewhat difficult or difficult to live on their current income, despite 49.9% of participants being employed. Thirteen percent of participants identified as Indigenous and 47.8% had children under 18 years old living at home. Nearly half (48.9%) resided in a large urban center, 27.5% in a medium-size city, and 23.6% in a rural area or small town. All but 20 women identified their partner as a man and most (72.3%) were not living with their abusive partner when they entered the study. Women’s plans for their abusive relationship varied: while half (51.3%) had ended the relationship and planned to stay separated, the next largest group (27.7%) were unsure about their plans. Of those who had separated from their partners, the average time since separating was less than 5 months. The level of abuse experienced by participants in the 6 months prior to the baseline survey was substantial with 82.5% experiencing severe combined abuse. The majority of women reported experiencing health problems that interfered with their daily lives including being nervous or uptight (88.5%), sad or depressed (90.0%), having fatigue or difficulty sleeping (91.8%), and experiencing pain (77.5%). There was between-group balance on participant baseline characteristics, suggesting that randomization was effective in preventing potential systematic biases in sampling that could have affected outcomes across the groups.

**Table 2:** Sample Characteristics by Intervention Group at Baseline

	Total *N* = 462		Non-Tailored *N* = 231		Tailored *N* = 231		
	n	M (SD)	n	M (SD)	n	M (SD)	p^d^
Age	414	34.61 (10.7)	208	34.39 (10.6)	206	34.84 (10.8)	.669
Months separated from partner (baseline)	266	4.77 (3.47)	129	5.01 (3.55)	137	4.55 (3.39)	.456
	*n*	%	*n*	%	*n*	%	p
Education							.287
No secondary school diploma	56	11.7	27	11.7	29	12.5	–
Secondary school diploma	82	17.7	35	15.2	47	20.3	–
Some post-secondary	148	32.0	72	31.2	76	32.9	–
Completed post-secondary	176	38.1	97	42.0	79	34.2	–
Employment							.646
Employed Full-Time	113	24.5	54	23.4	59	25.5	–
Employed Part-Time	116	25.1	62	26.8	54	23.4	
Unemployed	231	50.0	113	48.9	118	51.1	–
Missing	2	0.4	2	0.9	0	0	–
Difficulty Living on Current Income							.586
Not at all difficult	30	6.5	16	6.9	14	6.1	
Somewhat difficult/difficult	215	46.5	112	48.5	103	44.6	
Very/extremely difficult	217	47.0	103	44.6	114	49.4	
Indigenous Identity							.757
No	397	85.9	199	86.1	198	85.7	
Yes	62	13.4	31	13.4	31	13.4	
Missing	3	0.6	1	0.4	2	0.9	
Children < 18 years of age living at home							.514
No	241	52.2	117	50.6	124	53.7	
Yes	221	47.8	114	49.4	107	46.3	
Community of Residence							.420
Rural community or small town	109	23.6	52	22.5	57	24.7	
Med-Sized City	127	27.5	59	25.5	68	29.4	
Large Urban Center	226	48.9	120	51.9	106	45.9	
Partner’s Gender							.264
Man	442	95.7	223	96.5	219	94.8	
Other than man^a^	20	4.3	8	3.4	12	5.2	
Living with Abusive partner							.676
No^b^	334	72.3	165	71.4	169	73.2	
Yes	126	27.3	65	28.1	61	26.4	
Missing	2	0.4	1	0.4	1	0.4	
Plan for Relationship							.630
Plan to stay/plan to return	41	8.9	24	10.4	17	7.5	
Plan to leave	52	11.3	28	12.1	24	10.4	
Ended and plan to stay separated	237	51.3	115	49.8	122	52.8	
Unsure	128	27.7	63	27.3	65	28.1	
Missing	4	0.9	1	0.4	3	1.3	
Abuse Type in Previous 6 Months^c^							
Severe Combined Abuse	381	82.5	195	84.4	186	81.2	.364
Physical Abuse	395	85.5	191	82.7	204	88.7	.065
Emotional Abuse	458	99.1	228	99.1	230	99.6	.156
Harassment	364	78.8	182	78.8	182	79.5	.856
Self-Reported Health Problems							
“nervous” or “uptight”	409	88.5	201	87.0	208	90.0	.246
“sad” or “depressed”	416	90.0	209	90.5	207	89.6	.751
“fatigue” or “difficulty sleeping”	424	91.8	210	90.9	214	92.6	.399
“Pain (e.g. headaches, joint pain”)	358	77.5	179	77.5	179	78.2	1.000

^a^Inclusive of woman, trans woman, genderqueer, 2-spirited, no option that applies

^b^Inclusive of women who had separated and those who never lived with the abusive partner

^c^based on cut-scores for 4 subscales of the Composite Abuse Scale

^d^based on t-tests for continuous variables, ANOVA for categorical variables

### Primary and secondary outcomes

Table 3 presents the means and standard deviations across time on the primary and secondary outcomes. Both groups improved significantly over time on the primary outcomes of depression (*p* < .001) and PTSD symptoms (*p* < .001). However, the change over time did not differ between the tailored and non-tailored groups for either depression (*p* = .598) or PTSD (*p* = .269). A similar pattern was found for the secondary outcomes. Specifically, there was significant improvement over time in both groups on experiences of coercive control (*p* < .001), helpfulness of safety strategies (*p* < .001), confidence in making a safety plan for themselves (*p* < .001) and for their children (*p* = .023), and social support (*p* < .001) but the change across time did not differ between the two groups. Mastery decreased in both groups over time (*p* < .001), with no group differences in change over time observed. For the outcome of decisional conflict, immediately after a single use of the tool, women in both groups reported a significant decrease in all 4 aspects of decisional conflict (*p* < .001) but there were no differences over time between the groups for uncertainty (*p* = .316; ES = -0.08), feeling uninformed (*p* = .057; ES = -0.21), lack of values clarity (*p* = .423; ES = -0.10) or lack of support (*p* = .938; ES = 0.01).

**Table 3:** Longitudinal Changes in Primary and Secondary Outcomes by Study Arm

Outcomes	Non-Tailored Online Tool				Tailored Online Tool				Interaction *p*-value	Effect Size^a^
	Baseline	3 months	6 months	12 months	Baseline	3 months	6 months	12 months		
Depressive Symptoms	39.15 (21.34)	33.03 (20.38)	30.82 (20.31)	29.83 (21.26)	40.62 (21.00)	33.44 (20.79)	30.47 (22.15)	27.95 (22.50)	.598	−0.18
PTSD Symptoms	51.69 (14.46)	48.93 (14.41)	46.08 (15.49)	44.45 (15.81)	53.00 (14.24)	47.94 (14.91)	45.44 (16.40)	43.29 (16.82)	.269	−0.17
Experiences of Coercive Control	49.93 (9.37)	44.77 (11.93)	42.28 (14.12)	40.94 (14.69)	50.15 (8.80)	43.09 (11.66)	42.04 (14.15)	39.62 (15.73)	.645	−0.17
Helpfulness of safety strategies	3.23 (0.81)	3.29 (0.95)	3.40 (0.97)	3.54 (0.96)	3.21 (0.85)	3.34 (0.85)	3.50 (0.90)	3.55 (0.91)	.420	0.04
Confidence in safety planning for self	65.65 (26.87)	69.66 (23.33)	73.59 (23.76)	76.77 (22.32)	69.02 (23.56)	72.05 (23.87)	76.90 (21.79)	79.55 (21.94)	.927	−0.02
Confidence in safety planning for children	74.82 (29.55)	80.29 (25.73)	76.91 (28.86)	80.55 (24.85)	82.63 (25.62)	81.73 (25.12)	84.39 (21.16)	86.33 (22.39)	.266	−0.07
Mastery	20.87 (5.24)	18.15 (4.25)	19.09 (4.19)	19.97 (4.39)	20.85 (5.62)	18.79 (4.09)	19.42 (4.47)	19.91 (4.42)	.401	−0.01
Social Support	2.62 (0.96)	2.69 (0.96)	2.86 (1.05)	2.89 (1.06)	2.73 (1.06)	2.78 (105)	3.05 (1.11)	3.13 (1.13)	.627	0.13

^a^Effect size are Cohen’s d with change computed as (12-months – baseline), where d = (change in tailored – change in non-tailored)/baseline pooled sd

### Subgroup differences

**Fig. 2:**
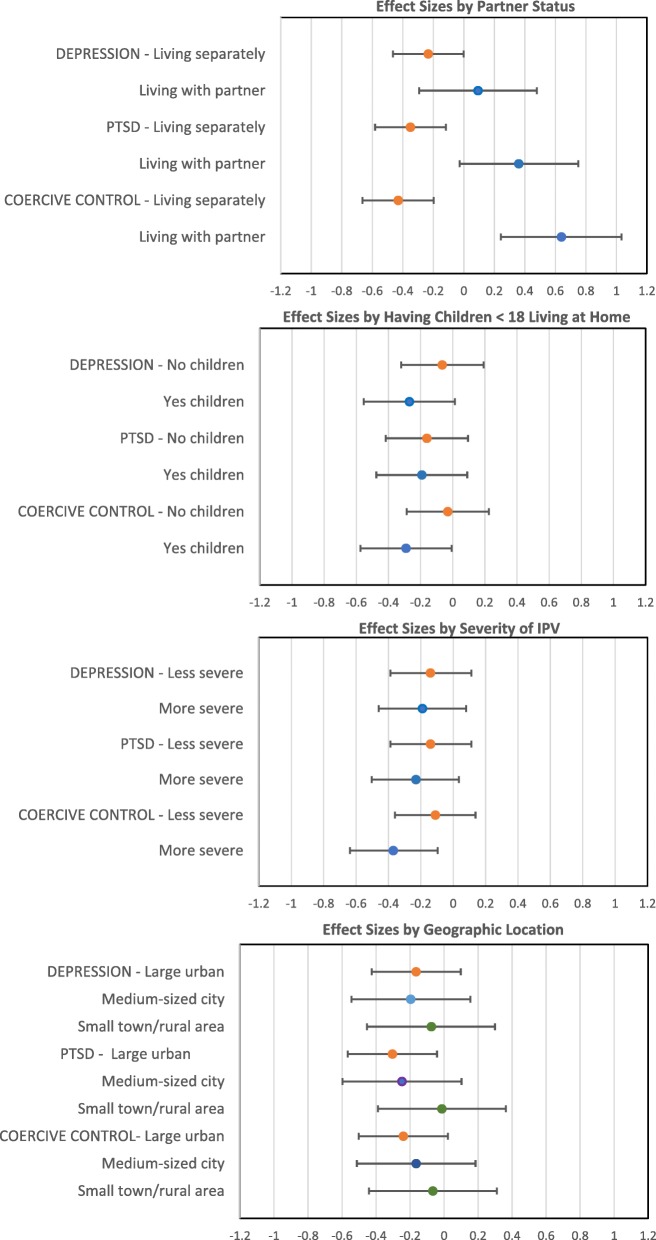
Cohen’s d effect sizes within a 95% CI of tailored versus non-tailored online intervention for depression, PTSD and coercive control by subgroups. Group A: living separately from or with partner; Group B: having or not having children < 18 living at home; Group C: Less severe or more severe IPV; Group 4: geographic location (large urban, medium-sized city or small town/rural area)

Consistent differential effects of the tailored and non-tailored online interventions were found for several subgroups of women (see Fig. 2). For women with children under the age of 18 living at home compared to those without children at home, the tailored intervention had a greater effect than the non-tailored version in reducing depression (ES = -0.27 vs ES = -0.06) and experiences of coercive control (ES = -0.29 vs − 0.03). The effect of the tailored versus non-tailored version was similar for women with and without children under 18 living at home on reduction in PTSD (ES = -0.19 vs ES = -0.16). For women reporting more severe violence at baseline compared to those reporting less severe violence, the tailored version also had greater effects than the non-tailored version in reducing PTSD (ES = -0.23 vs ES = -0.14) and experiences of coercive control (ES = -0.37 vs- 0.11). The effect was similar for women with more and less severe violence for depression (ES = -0.19 vs ES = -0.14). Differences were also noted across different geographic contexts; for women in large urban centers and medium-sized cities versus small towns/rural areas, the tailored version had greater effects than the non-tailored version in reducing depression (Large ES = -0.16, Medium ES = -0.20, Small/rural ES = -0.07), PTSD (Large ES = -0.30, Medium ES = -0.26, Small/rural ES = -0.01), and coercive control (Large ES = -0.24, Medium ES = -0.17, Small/rural ES = -0.07). Finally, compared to women who were living with a partner, those who were not living with a partner at baseline experienced a greater reduction in depression (ES = -0.23 vs ES = 0.09), PTSD (ES = -0.35 vs ES = 0.36), and experiences of coercive control (ES = -0.43 vs 0.64) when completing the tailored versus non-tailored version. In contrast, women who were living with an abusive partner at baseline versus those who were not living with a partner, showed a greater reduction in depression, PTSD and experiences of coercive control when completing the non-tailored version. A simplified summary of subgroup effects is provided in Table 4.

**Table 4:** Summary of Differential Benefits of the Tailored Online Intervention

Subgroup/Condition^a^	Outcome		
	Depression	PTSD	Coercive Control
Not living with Partner ^b^	x	x	x
Children < 18 living in the home	x		x
More Severe Abuse		x	x
Living in Large or medium-sized city	x	x	x

^a^based on baseline data

^b^women who were living with a partner at baseline benefitted from the non-tailored intervention for all 3 outcomes

### Benefits, safety, acceptability and potential harms

In the exit survey, women in both groups reported high levels of benefit, safety and acceptability, and low risk of harm associated with completing the intervention and participating in the study. For example, a high proportion of women agreed or strongly agreed that they gained something from the intervention (tailored 96.0%, non-tailored 93.8%), felt comfortable and safe (tailored 96.6%, non-tailored 95.3%), and would recommend it to other women (tailored 95.0%, non-tailored 90.0%). About one-quarter (tailored 29.3%, non-tailored 24.9%) reported that they felt anxious or upset when engaging with the tool, but most (tailored 92.5%, non-tailored 91.3%) also said they would have still taken part in the study. Indeed, some women in both groups submitted comments on their exit surveys indicating the tool had been “life changing” or “a life-line” that raised their awareness of risks and options and/or strengthened their confidence and resolve to deal with the challenges they were facing. However, women who completed the tailored intervention were more positive about the fit of the tool with their needs and concerns and were more likely to recommend it to other women (Table 5).

**Table 5:** Women’s Ratings of Benefits, Safety, Harms and Acceptability of Interventions and Participation by Group

Variable	Item	Tailored Group Ratings^a^		Non-Tailored Group Ratings^a^		p-value	Effect Size d’
		N	M (SD)	N	M (SD)		
Perceived Benefits	I gained something from completing the online tool	201	4.51 (.625)	209	4.45 (.699)	.380	0.09
Fit	The information in the online tool fit with my needs and concerns	201	4.28 (.756)	209	4.11 (.921)	.044	0.20
Safety	I felt comfortable and safe taking part	201	4.63 (.603)	209	4.59 (.723)	.511	0.06
Potential Harms	Working through the online tool made me very anxious or upset	201	3.22 (1.246)	209	3.33 (1.209)	.380	−0.09
Acceptability	If I had known what this study would be like, I would still have taken part	201	4.46 (.700)	207	4.35 (.798)	.159	0.15
Acceptability	I would recommend the online tool to other women	200	4.62 (.599)	209	4.47 (.766)	.038	0.22

^a^Response options: Strongly Disagree (1), Disagree (2), Neither agree or disagree (3), Agree (4), Strongly Agree (5)

Women in the tailored group also found the online tool significantly more helpful in preparing them to deal with abuse than women in the non-tailored tool group. Specifically, immediately after first use of the tool, women in the tailored group were more positive about the extent to which the tool helped them: recognize that safety decisions needed to be made (*p* = .061, ES = .18); think about the risks and benefits of each safety decision (*p* = .046, ES = .19); know which risks and benefits of safety decisions are important to them (*p* < .001, ES = .35); and know that safety decisions depend on what matters most to them (*p* = .004, ES = .28). The same pattern of results was noted in the 12-month survey, with women commenting on their exit surveys about the tailored components. For example, one woman wrote, “I was surprised when I found out that I am in the highest, most severe abusive risk category. It is so much worse than I was even able to explain”. Another wrote “Amazing to get an outside view of the risks and benefits in my own personal life and get tips on how to build myself”. Women in both groups also commented on how some of the survey questions (that were not part of the intervention) helped them think differently about their situation.

## Discussion

The results of this study extend existing trial evidence from the U. S, New Zealand and Australia supporting the safety, acceptability, and low risk of harm of online safety and health interventions to Canadian women. Consistent with those studies, our results also show that women in both intervention groups (tailored and non-tailored) improved on primary and secondary outcomes over time. Importantly, our findings also provide new evidence about the differential benefits of a complex online safety and health intervention for specific groups of women and contribute new insights that help to create a more contextualized and nuanced understanding of intervention processes and impacts. Adopting research approaches that are capable of evaluating differential effects and processes, as well as group differences on outcomes, is essential for conducting rigorous evaluations of complex interventions, such as *iCAN*.

Our results do not support the overall effectiveness of the tailored online safety and health intervention when compared to a non-tailored version of the tool. Our original intent was to compare the tailored intervention to a true control condition, but during the development phase, we realized that, on ethical and safety grounds, this was not possible. Thus, although we proposed an RCT with a true control group, this study really compared two interventions, with the results supporting similar parallel trends in improvement across groups. Given that randomization achieved balance between the groups and based on insights from our process evaluation, it is plausible that the lack of differences in outcomes between the study arms is due, at least in part, to: a) similarity in the intervention content (one more in-depth and tailored, the other simpler and not personalized), both of which were highly rated by the women; b) the non-judgemental, inclusive and supportive ‘tone’ of each intervention, such that women in both groups reported that they felt respected, validated and heard, features that are important in supporting women’s healing from trauma and abuse; and c) the likelihood that the study measures acted as an intervention that raised women’s awareness about options for managing the violence and mental health problems (the primary study outcomes). As reported elsewhere [40], women in both groups noted that these “background questions” were an important and *helpful* part of the intervention. The lack of differences by study arm is consistent with the overall pattern of results found in 3 completed trials that also tested versions of a tailored intervention with the same core components, modified to fit different countries and contexts, against a non-tailored intervention [25, 37–39].

That a high proportion of women in both groups reported that they found the intervention safe, acceptable, and beneficial with no evidence of harms reinforces the potential usefulness of both online tools for women. Indeed, findings from our process evaluation provide important insights about the mechanisms that could lead to improvements in women’s mental health. Specifically, women noted that the online intervention provided time and space to consider their risks, options and priorities and strengthened their confidence, control and commitment (aspects of positive mental health) to address the violence in ways that were best for them [40]. Ironically, increased awareness among women may have also contributed to the small but statistically significant decrease in mastery (sense of control) over time in both groups. Given that factors such as health problems, ongoing violence and the costs of getting help have been shown to erode women’s sense of control in the context of IPV, particularly post-separation [7], similar decreases in mastery observed across groups may also be unrelated to the study.

Further, while women in both groups reported benefits, those in the tailored group reported that it was a ‘better fit’ with their needs and were more likely to recommend it to other women, suggesting that tailoring or personalizing these types of interventions may still be important. While these results provide further support for the importance of personalizing online interventions, they do not address the challenges of doing this in the context of significant complexity, given women’s varied priorities, needs and resources. Women who completed the tailored version were given an opportunity to modify their action plans, but the initial information provided to them was based on a set of assumptions identified by the research team. Given that women who have lived through violence are often very resourceful and resilient [63, 64], developing approaches that enhance self-tailoring by women themselves may be a more effective alternative. This requires further study.

Consistent with the methodological literature on the evaluation of complex interventions [14], we sought to examine more than global effects by study arm to also understand who might most benefit from the tailored intervention and what might explain these effects. Although the subgroup analyses are not statistically powered, comparing the effect sizes across categories within a subgroup provides valuable information about the heterogeneity of treatment effects [65]. Indeed, the small effect sizes observed in our main analysis are consistent with our finding that the tailored online intervention is not equally effective across groups. In this context, the subgroup analyses allow us to provide a more comprehensive explanation about the impact of the intervention. Specifically, our results underscore the differential benefits of the tailored intervention on mental health and experiences of coercive control for 4 groups of women: those with children under the age of 18 living with them, who were not living with a partner, who experienced more severe violence, and who were living in medium or large urban settings. Importantly, these results also yield insights about what could be modified to improve effectiveness of the tailored online intervention for women who did not benefit as much.

For *women with children under age of 18*, the tailored version was more effective than the non-tailored version in reducing symptoms of depression and PTSD, and women’s experiences of coercive control, than it was for women who did not have children under the age of 18. Women who are parenting children often prioritize their children’s safety, health and well-being, sometimes over their own [66, 67]. In this context, they may be more compelled to address the violence because of the risks to children. A tailored plan that helps women make a cognitive connection between their children’s safety and well-being and their own health and well-being may be more helpful in supporting women’s actions than a brief static tool focussed primarily on emergency planning. These findings are important given that the mental health and safety of mothers is critical to their own well-being and functioning, effectiveness of parenting, and ability to contribute to society [68–70].

The majority of women (72.3%) who participated in this study were *not living with an abusive partner* at baseline. For these women, the tailored version was more effective than the non-tailored version in reducing symptoms of depression, and women’s experiences of coercive control as compared to women who were living with a partner. Intensive, tailored strategies that broadly address women’s safety and quality of life may be *more appropriate* for women who are no longer living with a partner and are in the transition of “moving on”; in this context, women’s priorities are linked to and extend beyond safety and they are often more ready to begin addressing multiple issues, such as health and well-being and economic issues, that become important as they plan for the future [7, 13]. Importantly, post-separation abuse [71] and ongoing health problems are common for these women, yet violence services often focus on times of crisis and not on addressing longer-term needs. A tailored online tool such as *iCAN* is a low-cost option to fill this gap in ways that could complement and, potentially, extend existing services.

The finding that women who were living with an abusive partner at baseline benefitted more from the non-tailored intervention was unexpected*.* Focussed, direct strategies for improving safety in emergency or crisis situations may fit better with the immediate concerns of women who are dealing with day-to-day survival. The level and complexity of information and options presented in the tailored online intervention may have been overwhelming and unhelpful for this subgroup of women. This finding further supports the notion that ‘one size fits all’ interventions risk not adequately meeting the unique needs of women and reinforces the need to prioritize both usability and choice in the design and testing phases of these types of online interventions.

That the tailored intervention was more effective than the non-tailored tool for women who reported more severe violence at study entry is critically important since these women are known to face the greatest risks of harm and poor mental health [72]. Indeed, in this study, more severe abuse was associated with higher PTSD symptoms and coercive control. Having time in a private space to reflect on their experiences and get personalized feedback on their risks and safety strategies may have been particularly validating and impactful given the level of ongoing threat these women were facing. More severe violence has also been associated with greater isolation and with more significant social and economic impacts [34, 35]. Our results suggest that the tailored online intervention has specific benefits and may be an effective means of safely engaging groups of women who may be harder to reach with conventional services, including those women dealing with both more severe violence and greater economic and social disadvantages.

For women living in both *medium-size cities* and *larger urban centers* at baseline, the tailored online tool was more effective than the generic tool in reducing symptoms of depression and PTSD and reducing experiences of coercive control than it was for women living in rural and small-town settings. As reported elsewhere [40], in qualitative interviews and exit comments women described using the tailored tool in conjunction with other services as part of their help-seeking. Larger centers are more likely to have services and resources that women can access to deal with violence and related issues; research has documented the unique barriers faced by rural women that make it particularly difficult to deal with IPV, including public visibility, lack of privacy, few appropriate local support services and perceived lack of options for staying safe [73–75], concurrent with increased risk of homicide from their abusive partners [75, 76]. It is possible that we failed to adequately personalize the messages in the action plan to reflect their unique needs and experiences (e.g., strategies had an unrecognized ‘urban bias’, suggesting that women seek out services that might not exist). There is a need to further explore the particular needs and experiences of women living in rural and small-town settings with respect to what was helpful and not helpful about the online tool and how it could be strengthened to better fit with their needs. A more in-depth analysis of the mechanisms that explain interventions effects is also warranted, inclusive of whether and how women’s access to services recommended in the online intervention is related to key outcomes.

### Limitations

Participation in this trial was limited to adult women who could participate in English, who had safe access to a computer and email address and who had experienced recent IPV. In spite of this, we recruited a relatively diverse volunteer sample of women who were interested in engaging with an online intervention, inclusive of women who faced significant barriers to support and are often under-represented in research. Indeed, representation of Indigenous women exceeded population rates (13.4% compared to 4% in the Canadian population) [77], while the participation of women living in rural communities and small towns was substantial, although somewhat less than in the Canadian population (23.6% compared to 30.5%) [78]. Although we adopted many strategies to recruit women with partners other than men, we had limited success (5% of overall sample), limiting generalizability of the results to this group. While we make no claim that the study sample is representative of the population of Canadian women who have experienced IPV, the diversity of the sample enhances the applicability of our findings to women from diverse backgrounds.

Although *iCAN* was developed in collaboration with women who would be end-users and domestic violence, health and social service professionals, changes to this tailored online intervention may still be needed to improve its fit for some groups of women and/or to allow women to self-tailor their action plans even more. Women who had been separated from an abusive partner for more than 12 months were ineligible for this trial, yet their interest in participating was high. Given that dealing with IPV and the negative consequences of IPV is often a long-term process, and that women who were not living with an abusive partner benefitted from the tailored intervention, the potential relevance of *iCAN* for women who have been separated for longer than 1 year should be considered. However, this needs further study.

As previously noted and consistent with previous research, it is also possible that the baseline survey measures (both arms) could have biased the findings, as the questions themselves potentially functioned as an intervention [79], increasing, for example, women’s self-awareness of their IPV experiences, safety actions and mental health. Further, there was no true control group, as it is was unethical to provide ‘nothing’ to women. In future studies, it is important to consider the most appropriate designs for testing complex interventions like *iCAN* [80], and to consider the potential influence of baseline measures on outcomes. The incorporation of process-oriented data from women in this trial via exit surveys, along with the subgroup analysis, resulted in important insights that would not be possible if the focus had been on measuring primary and secondary outcomes alone.

## Conclusion

Given women’s positive perceptions, lack of evidence of harms and demonstrated effectiveness for specific groups of women, we argue that *iCAN* is a promising intervention, with differential benefits for women’s mental health and experiences of coercive control among those not living with an abusive partner, living with children, experiencing more severe violence, and living in medium to large urban settings. It is important to acknowledge that online interventions such as this may not be appealing to all women and that they should not be seen as a replacement for services but as a resource for women and for providers working with women. Additional findings from qualitative interview data may shed light on strategies for strengthening the intervention and improving its impacts for a greater number of women.

## Trial status

Completed.

## Data Availability

The data used in these analysis are not publicly available and will not be shared as they contain information that could compromise research participant safety and violate the conditions under which informed consent was obtained.

## Abbreviations

DA: Danger Assessment
iCAN: iCAN Plan 4 Safety
IPV: Intimate partner violence
PTSD: Post-traumatic stress disorder
RA: Research assistant
